# Antioxidant activities in relation to the transport of heavy metals from the soil to different parts of *Betula pendula* (Roth.)

**DOI:** 10.1186/s13036-022-00322-8

**Published:** 2023-03-06

**Authors:** Iwona Makuch-Pietraś, Dorota Grabek-Lejko, Anna Górka, Idalia Kasprzyk

**Affiliations:** 1 Department of Nature Conservation and Landscape Ecology, Institute of Agricultural Science, Land Management and Environmental Protection, College of Natural Sciences, University of Rzeszów, Zelwerowicza 4, 35-601 Rzeszów, Poland; 2 Department of Bioenergetics, Food Analysis and Microbiology, Institute of Food Technology and Nutrition, College of Natural Sciences, University of Rzeszów, Zelwerowicza 4, 35-601 Rzeszów, Poland; 3 Department of Biotechnology, Institute of Biology and Biotechnology, College of Natural Sciences, University of Rzeszów, Pigonia 1, 35-310 Rzeszów, Poland; 4 Department of Biology, Institute of Biology and Biotechnology, College of Natural Sciences, University of Rzeszów, Pigonia 1, 35-310 Rzeszów, Poland

**Keywords:** New ecotoxicological indicator, Antioxidants, Stress, Silver birch

## Abstract

**Background:**

Birch is a tree with a common occurrence in the environment and its organs are used in the form of herbal material. An important aspect of this study is birch pollen, which is a problem for allergy sufferers, and due to a variety of environmental conditions, its allergenicity may increase. Among the organs studied, inflorescences deserve attention, which, as seen from an overview of the literature, are analysed for the content of heavy metals for the first time in this study.

**Results:**

This paper investigated the relationship between antioxidant properties and the content of heavy metals (Cu, Zn, Cd, Pb, Ni and Cr) as the plant's response to stress, taking into account both the vegetative and generative organs of the tree *Betula pendula.* While studying the accumulation of elements in individual organs, the research was extended to include the aspect of different environmental conditions, reflected in two soil types of differing physicochemical properties: sandy and silty soils. In order to thoroughly analyse the transport of the studied heavy metals from the soil to individual organs (leaves, inflorescences and pollen), ecotoxicological indicators were used. A modified translocation factor (TF) index into sTF (sap translocation factor) was presented as a novelty in research, calculated based on the content of selected heavy metals in the sap flowing to individual birch organs. This allowed for a more complete description of the transport of elements in the aerial parts of plants, indicating the accumulation of zinc and cadmium, especially in leaves. Among the studied environmental conditions which may affect the accumulation of heavy metals, sandy soil is of particular significance, conditioning lower pH values, among other things. However, analysis of the reaction of birch to the conditions of the soil environment and the content of heavy metals, based on antioxidant properties, demonstrated an evident reaction to stress, but without an unambiguous response among the studied vegetative and generative organs.

**Conclusions:**

As birch is a plant with wide utility values, monitoring studies are advisable to exclude the risk of accumulation of heavy metals in its organs, and for this purpose it may be useful to use the sTF indicator and assess the antioxidant potential.

**Supplementary Information:**

The online version contains supplementary material available at 10.1186/s13036-022-00322-8.

## Introduction

In the literature on the subject, the term 'heavy metals' is understood as those elements whose excess in the environment is harmful to living organisms. Apart from high-density metals, this group also includes aluminium and arsenic, metals with lower density. Heavy metals present in the environment can be of natural origin (weathering of rocks, volcanic eruptions) or anthropogenic. In the environment, they are a natural component of the earth's crust; however, diverse types of anthropogenic activity lead to disturbance in geochemical cycles, resulting in the accumulation of metal in relation to background levels [1–3]. Soil heavy metal pollution can come from a variety of sources, both natural sources and anthropogenic ones such as industry, fertilization, etc. This issue is important from an ecological, toxicological and health point of view. Increased content of heavy metals in the soil can be the reason for their accumulation in plants. However, their bioavailability depends on both environmental and physiological factors of the plant itself [3–5]. Among the mentioned environmental parameters affecting the accumulation and bioavailability of metals is soil texture. The most important role in retaining heavy metals in the soil is played by the content of the clay fraction in soil texture. In the case of sandy soils, the low content of the clay fraction leads to greater mobility of heavy metals, which makes them able to move both deep into the profile and greatly facilitates their bioavailability for plants [2, 3, 6]. The clay fraction, like organic matter, has a colloidal character and a high specific surface area, which allows the concentration of heavy metals and their solubility by immobilization to be monitored [6, 7]. The content of clay fraction can also affect the pH of the soil, which is mentioned in many works in the literature, as the main factor influencing the greater availability of elements with high soil acidification [2, 3, 6–8]. In addition, environmental factors include the influence of the heavy metal itself and its form present in the soil, followed by the possibility of penetration into the plant under appropriate soil conditions [2, 3, 6–9]. To a large extent, the impact of heavy metals depends on their properties, concentration in the environment, and the form present; free ions are taken up the fastest, while metals occurring in the form of complexes are released, but only with the help of active substances derived from plants [6, 10]. It is worth noting the properties of some metals (Cu and Zn), which in small amounts have a stimulating effect, constituting microelements which are an indispensable component necessary for the proper course of metabolic processes [2, 11]. A huge role in the process of uptake and accumulation of metals is also played by the plant itself. The process of accumulation in the plant takes place in three stages: an increase in the mobility of metal ions, uptake, then transport in the plant to various organs [10] However, there is a tendency to accumulate in the roots of plants, and then in the stems and leaves [10, 11]. The smallest accumulation is shown by flowers and then seeds [11]. The effect of toxicity on plants, manifested by deterioration of the condition of the plant and hampered growth, is caused only by a large accumulation of heavy metal [11, 12].

In the assessment of heavy metal interactions, useful tools for determining both soil contamination and also the bioavailability of metals for the plant as well as transport and accumulation are a series of quite extensively developed and discussed indicators [1, 5, 13, 14]. Among the wide range of indicators, one can choose those that fully characterise the geochemical cycle, indicating the most important elements of it prone to accumulation [14].

In tracing the accumulation of heavy metals in the plant, as well as in monitoring studies, trees are the most useful. The choice of trees is greatly influenced by their natural occurrence in industrial regions and in areas with strong urbanization, moreover, in areas where there are soils characterised by worse properties. An additional advantage of the use of trees is the large amount of biomass and the ability to analyse the transport of heavy metals to various plant organs and their preferences for accumulation [5, 15, 16]. Of great importance when choosing the right species for research is resistance to pollution, the popularity of the species, as well as the specificity of the organs selected for the experiment [5, 17]. An example of a tree with the above characteristics is birch *Betula pendula*, the use of which in research on the content of heavy metals has already been described in many works in the literature [1, 5, 15, 16, 18]. As indicated by Petrushkevych and Korshykov [18], research has focused mainly on roots, stems or leaves. Among the studies conducted on the species *B. pendula*, a large number also focus on sap, which is a product of increasing interest to consumers, thus requiring greater attention to quality [19]. On the other hand, little attention is paid to the influence of heavy metals and their accumulation in the generative organs, catkins and pollen, focusing attention rather on the impact of atmospheric pollutants [18, 20–22]. The influence of heavy metals on the generative organs will have the greatest impact during the growth and development of the plant, and thus on reproductive capacity [23, 24].

Excessive amounts of heavy metals have toxic effects on plants and their effects depend on the metal, concentration values, and tolerance of the plant. The increased content of toxic substances in plant tissues triggers a cascade of reactions, including the formation of an excessive amount of reactive oxygen species. Plants have developed some efficient mechanisms to scavenge ROS, including enzymatic and non-enzymatic ones [13, 25, 26]. Among these mechanisms are antioxidants. The term “antioxidant” refers to a class of compounds that protect the cell from damage caused by exposure to certain highly reactive species such as ROS and that are responsible for removing, neutralising, and scavenging ROS. Phenolic compounds are characterised by having various functions in plants, highlighting their antioxidant capacity observed under different stress conditions. Exposure to heavy metals generates reactive oxygen species (ROS) and increases the production of phenolics in plants. Phenols act as chelating metals through hydroxyl and carboxyl groups and also inhibit lipid peroxidation by trapping alkoxyl radicals [27].

Plant response on heavy metal contamination and possible toxic metal accumulation is very important from the consumer's point of view. Birch, in particular silver birch, has traditionally been important in many countries where all of the plant parts have been utilised for various medicinal purposes [28]. *B. pendula* has been used to treat urinary disorders, infections, inflammations, rheumatism, and skin diseases. Oil derived from birch buds is used as a tonic and antiseptic in cosmetic products [28]. Moreover, birch sap is a traditional beverage in boreal and hemiboreal regions of the northern hemisphere (harvested in spring, especially in Belarus, Estonia, Finland, Latvia, Lithuania, Poland, Romania, Russia, and Ukraine). Birch sap is used as a birch sap beverage flavoured with fruit, as birch syrup, and in cosmetics for skin and hair [19, 29]. Birch is a species with a strong link to human well-being and health. Its pollen has negative impact on people sensitive to it as an allergen, and if contaminated by heavy metals could present higher allergenicity [30]. In the European population, the prevalence of birch pollen sensitization ranges from 8 to 16% [31].

Due to the above, it was reasonable to perform a number of analyses regarding the content of heavy metals in various parts of birch and their bioavailability. This allowed us to verify several theses: the metal content in the soil depends on the physicochemical properties of the soil and the soil textural types. For each element, its content, bioavailability and translocation in the sap, vegetative, and generative organs of birch will vary. It was assumed that the higher accumulation of toxic metals in different parts of birch trees would result in the higher antioxidant potential and phenolic content as a response to heavy metal stress. The results of the research will allow us to answer the question of which birch organs accumulate the most heavy metals and thus experience a reduction in their use value (medicinal, culinary). An important question that was posed was whether the transfer and accumulation of metals to pollen is so large that they can be a potential factor increasing its allergenicity.

## Materials and methods

### Study area

**Fig. 1:**
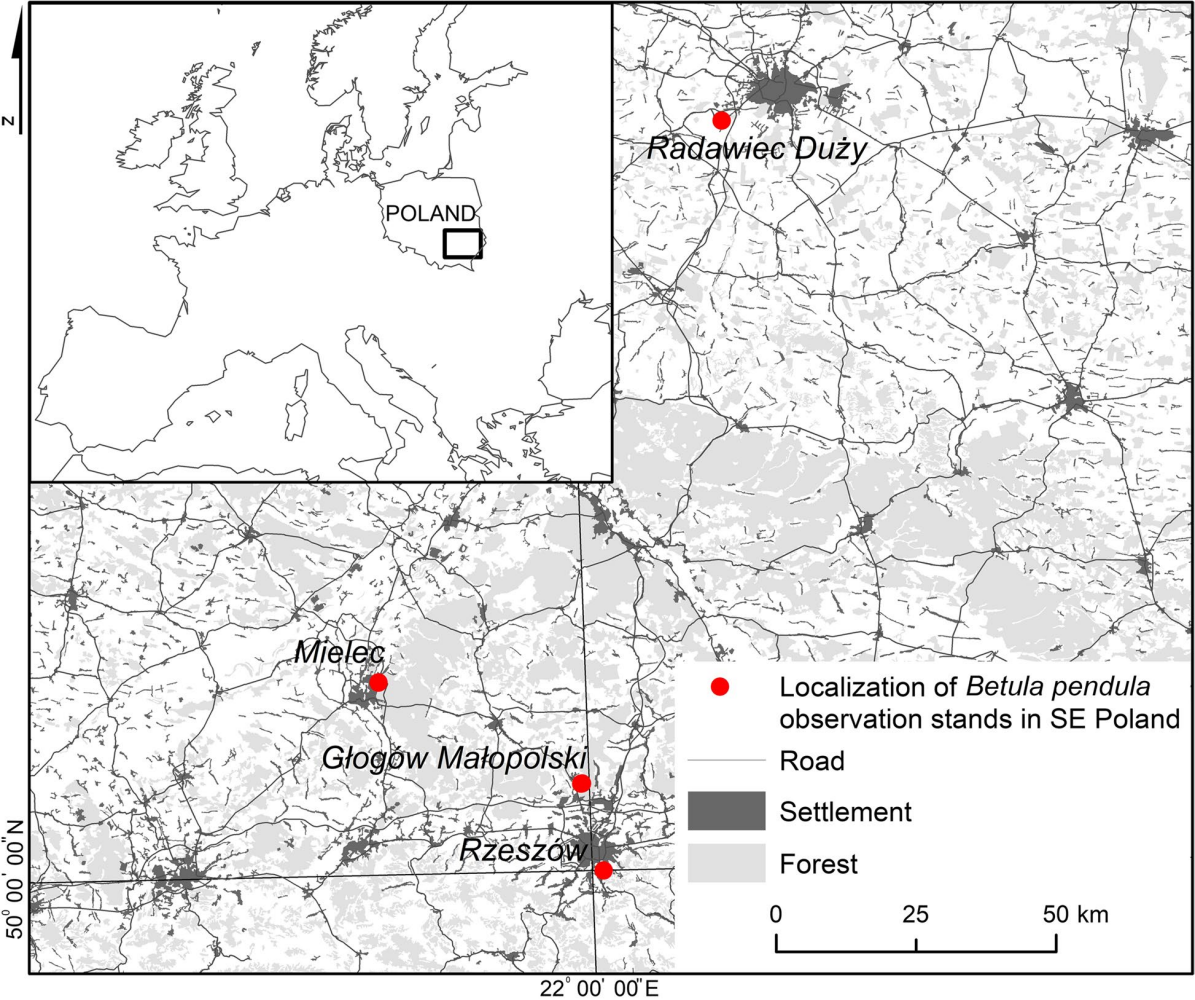
Localization of sampling sites

The study was conducted at 4 sites in southeast Poland in 2019 (Fig. 1). Based on the Geochemical Atlas of Poland [32], it was assumed that these sites should differ in pollution of heavy metals and in soil texture (the particle size composition of soils). There were two sites with sandy soils: Mielec (MI), with heavy metal contamination, and Głogów Małopolski (GM), which is characterised by rather HM low contamination. The same scheme was used on 2 sites with silty soils, where Rzeszów (RZ) was determined to be more polluted with heavy metals than Radawiec Duży (RD). The sites differ in topography, type of land use, and degree of air pollution. RZ is the largest city, followed by MI and GM. Among the described sites, MI stands out as a much more developed city in terms of industry, the result of which has been the creation of a special economic zone. RD is located near the city of Lublin and is the smallest locality, dominated by agricultural land.

### The object of the study

*Betula pendula* Roth. (silver birch) is a deciduous tree of wide distribution in warm and cold temperate climate of the northern chemisphere. It is a common component of numerous forest communities, including urban forests and parks. Because of its low habitat requirements and relative resistance to air pollution, silver birch is considered a pioneering plant. It prefers dry, sandy soils and sunny sites. In a warm temperate climate, it blooms from the second half of April until the beginning of May, and male catkins, forming in August and September of the previous year, produce enormous amounts of pollen known to be a strong allergen [33]. Its leaves, bark, and sap are commonly used in herbal medicine [34, 35].

### Material samples and preparation

*B. pendula* is frequently observed in the area of the study. Five silver birch trees along the transects were chosen at each study site. Sap (Sa), pollen (P), leaves (L) and catkins before (C_1_) and after (C_2_) anthesis were collected from the same trees. Birch catkins before opening, as well as birch sap were collected at the same time in February and March 2019. In order to collect sap, at a height of 30 cm of the trunk, small, 8 mm diameter holes were drilled with using a cordless drill and a carbide, feather drill bit, then, a plastic pipe (15 cm long and 8 mm in diameter) was put into the hole. Sap was directly collected from the pipe into the sterile plastic tubes (volume of 50 ml), transport to the laboratory, then filtered with cellulose filters (pore size—0.45 um) and frozen at -80 °C till analysis [29], but leaves, catkins after opening, and pollen were collected in April 2019. To collect pollen in sterile conditions, branches were transported to the laboratory and mature pollen grains were naturally released from anthers. After that, the catkins and leaves were dried in the dark at room temperature and then ground. Soil (So) samples were taken from rooting zone near each tree. The soil material was air dried and passed through a 2 mm mesh sieve. The physicochemical properties of the soil material were determined using the following methods. The particle size distribution was determined using the Bouycous and Cassagrande areometric method, modified by Prószyński. Soil fractions and formations were identified according to PTGleb [36]. The soil pH was determined potentiometrically in water and in a 1 M KCl solution. The organic carbon content was measured using the Tiurin method [37].

### Heavy-metal content determination

The analytical procedure for the determination of the mineral elements involved using flame atomic absorption spectrometry (Contraa 700, Analytik Jena) as well as a graphite furnace. 1 ml of birch sap, 0.5 g of other birch samples, 0.2 g of soil were transferred to a teflon beaker with 8 ml of concentrated nitric acid and mineralised using microwave methods (Ethos One Milestone). After mineralisation, the samples were filled up to 10 ml with water and used for the detection of heavy metal (HM) mineral elements. For the measurement of zinc (Zn), copper (Cu), nickel (Ni), chromium (Cr), cadmium (Cd), and lead (Pb), the samples were diluted to obtain a sensitivity within the optimum working range. A daily calibration curve was performed for the quantification of the different mineral elements. The accuracy of the method was confirmed by analysis of several certified reference materials such as Standard Reference Material 1570a—Trace Elements in Spinach Leaves; NCS ZC 73,005 Soil (Soil Certificate of Certified Reference Materials approved by the China National Analysis Center in Beijing, China), standard: ICP multi-element standard IV (Certipur Certified Reference Material, Merck). A software package (ASpect CS software) was used to calculate the mineral concentration.

### Ecotoxicological indicators

Based on the obtained results, indicators have been calculated, the use of which is an important tool when describing the relationships between the soil and the plant. They also allow us to compare the transfer and bioaccumulation of the tested heavy metals. The following indicators were used in the work:

*MR (Mobility ratio)*—corresponding to BAC (Biological accumulation coefficient), which explains the transport from the soil to the individual organs of the plants, calculated on the basis of the metal content of the above ground plant part (C_plant_) to the soil content (C_soil_) [5].

$$\tt MR=C_{plant}/C_{soil}$$

*sTF (Sap translocation factor)* – describing transport only in the plant; it has been modified for research purposes and has been calculated on the basis of the metal content in the aerial parts of plants (C_plant_) to the content in birch sap (C_sap_) [5 with modifications].

$$\tt sTF=C_{plant}/C_{sap}$$

*PI*_*i*_* (Single soil pollution index)*—comparison of the current concentration of a given mineral (i) in the soil (C_soil_) with the geochemical background (B_m_); [14]; the following values were adopted as B_m_: Zn – 67 mg/kg; Cu – 39 mg/kg; Cr – 69 mg/kg; Ni – 55 mg/kg; Pb – 17 mg/kg; Cd – 0.1 mg/kg [38, 39].

$$\tt PIi=C_{soil}/Bm$$

Any result greater than 1 means that the metal content in the test sample was higher than that considered typical [14].

*PI total (Sum of soil pollution indexes)*—is the sum of the indices of individual heavy metals [14]

$$\tt {\mathrm{PI}=\Sigma}_{i}PI$$

### Antioxidant Activities determination

The antioxidant potential is also measured by the content of secondary metabolites in the plant. Antioxidant properties were measured using two methods: FRAP (based on reducing Fe3 + to Fe2 + ions, which react with TPTZ (2,4,6-tris(2-pyridyl)-1,3,5-triazine) and DPPH based on scavenging free stable radical DPPH [40]. The content of polyphenols and flavonoids was determined.

### Preparation of birch extracts

One g of each sample was immersed in 10 ml of extraction solution, composed of methanol and water (80:20, v/v). The extraction was carried out at room temperature for 24 h. The extract solutions of were recovered by filtration using Whatman Filter paper, 0.45 µm (Whatman, GE Healthcare, Wauwatosa, WI) and immediately used for analysis. Birch sap was kept frozen at -20^°^ C until analysis.

### Ferric reducing antioxidant power assay (FRAP)

A manual assay was used based on the methodology of Benzie and Strain [41]. FRAP reagent was freshly prepared with 1 mM 2,4,6-tripyridyl-2-triazine (TPTZ), 2 mM ferric chloride in 0.25 M sodium acetate buffer, pH 3.6. Aliquots (0.02 ml) of properly dissolved and prepared samples were added to 0.18 ml of FRAP reagent in a 96-well microplate, incubated for 10 min at room temperature and measured spectrophotometrically at 593 nm. A standard Trolox solution was used for the calibration curve and the results were expressed as µmoles of Trolox equivalent per 1 g of sample or per 1 L of birch sap.

### DPPH assay (Radical Scavenging Activity).

The antiradical activity of different birch samples was determined using the synthetic free radical 2, 2-diphenyl-1-picrylhydrazil DPPH method according to Dżugan et al., [42], with some modifications. Briefly, the extracts of the birch samples were dissolved 50 times in methanol (final concentration 2 mg/ml), the birch sap was not dissolved and 0.02 ml of these mixtures were mixed with 0.18 ml of 0.1 mM DPPH (Sigma Aldrich Co., St. Louis, MO, USA) solution in methanol (POCH, Gliwice) and left in the dark at room temperature for 30 min. Then the decrease in absorbance was measured spectrophotometrically at 517 nm using methanol as a blank. Trolox (Sigma Aldrich Co., St. Louis, MO, USA) and quercetin (Sigma Aldrich Co., St. Louis, MO, USA) at a concentration of 0.1–100 µg/ml were used as a positive control.

The radical scavenging activity (A%) was calculated using the following formula:

$$\begin{array}{c}\mathrm A\%=(({\mathrm A}_0-{\mathrm A}_{\mathrm a})/{\mathrm A}_0)\mathrm x100\\{\mathrm A}_{\mathrm a}-\mathrm{the}\;\mathrm{absorbance}\;\mathrm{of}\;\mathrm{the}\;\mathrm{study}\;\mathrm{sample}\\{\mathrm A}_0-\mathrm{the}\;\mathrm{absorbance}\;\mathrm{of}\;\mathrm{the}\;\mathrm{control}\;\mathrm{sample}\end{array}$$

The positive control for the DPPH assay: Trolox at concentration 10 and 50 µg/mL showed 11.40% and 54.63% inhibition, respectively; quercetin at concentration 10 and 50 µg/mL showed 15.52% and 66.72% inhibition, respectively.

### Determination of total phenolic compounds

The total phenolic compounds in the birch extracts and the birch sap were determined with the Folin-Ciocalteu reagent according to the method of Slinkard and Singleton [43] using gallic acid as the standard phenolic compound. Briefly, 0.1 ml of the analysed extracts were mixed with 0.5 ml of Folin-Ciocalteu reagent and incubated for 5 min at room temperature. Then 3 ml of Na_2_CO_3_ was added and the probes were incubated for 90 min. After that, the absorbance at 725 nm was measured against water as a blank. Gallic acid (GAE) was used for the calibration curve and the results were expressed as mg of GAE per 1 g of sample or per 1 L of birch sap.

### Total flavonoid content

The total flavonoid content was determined using the method described by Meda et al. [44] with some modifications. 0.1 ml of 2% aluminium trichloride (AlCl_3_) in methanol was mixed with the same volume of extract solution. The mixture was incubated for 10 min at room temperature. The absorbance was measured at 415 nm against blank samples (methanol). Rutin (Sigma Aldrich Cl., St. Louis, MO, USA) was used for the calibration curve. The results were expressed as micrograms of rutin per g of product and micrograms of rutin per 1 L of birch sap.

## Data analysis

Due to the lack of normality of the data (the Shapiro–Wilk test) and / or homogeneity of variance (Brown-Forsythe test), nonparametric tests were used for two and more samples, U Mann–Whitney and Kruskal–Wallis tests, respectively. For more than two groups, detailed pairwise comparisons were made using Dunn's test. Soil texture group (sandy, silty), sites (RD, RZ, GM, MI) or type of plant material were the grouping variables (Sa, L, P, C_1_, C_2_). The non-parametric rank Spearman correlation test was applied to examine the relationships between pairs of variables. The relationships between the concentrations of minerals in plants and soil and antioxidant activities were detected using multiple regression. The results were regression formulas with multiple correlation coefficients and multiple determination coefficients (R^2^). For all statistical tests, the level of significance was α ≤ 0.05.

To check the relationships between the selected soil parameters (explanatory environmental variables) and the content of chemical elements in soil and plant, their mobile ratio and sTF separately (the explained variables) in the context of the soil texture, a multidimensional constrained analysis was applied. As the gradient was 0.1 SD units long, therefore, a linear method, redundant analysis (RDA), was applied. The cumulative percentage of explained variation (PEV) and the *p*-value for pseudo-F were given for four axes. The results were illustrated in the ordination triplots, where three types of scores were taken into account: the soil texture (sandy, silty), the soil parameters (the independent variables), and the plant materials (the explained variables). The two last mentioned were shown as vectors, while the nominal variable (the soil texture) was shown as points. The length of the vector reflects the usefulness of each independent variable in explaining the variability of the dependent variables. The longest vector means that it is the variable that contributes the most to explaining the variability in the ordination space. The angle of vectors indicates the correlation of variables, and the smaller the angle, the greater the positive relationship. An angle above 90° can be interpreted as a negative correlation.

## Results

### Physicochemical characterisation of the soils

**Fig. 2:**
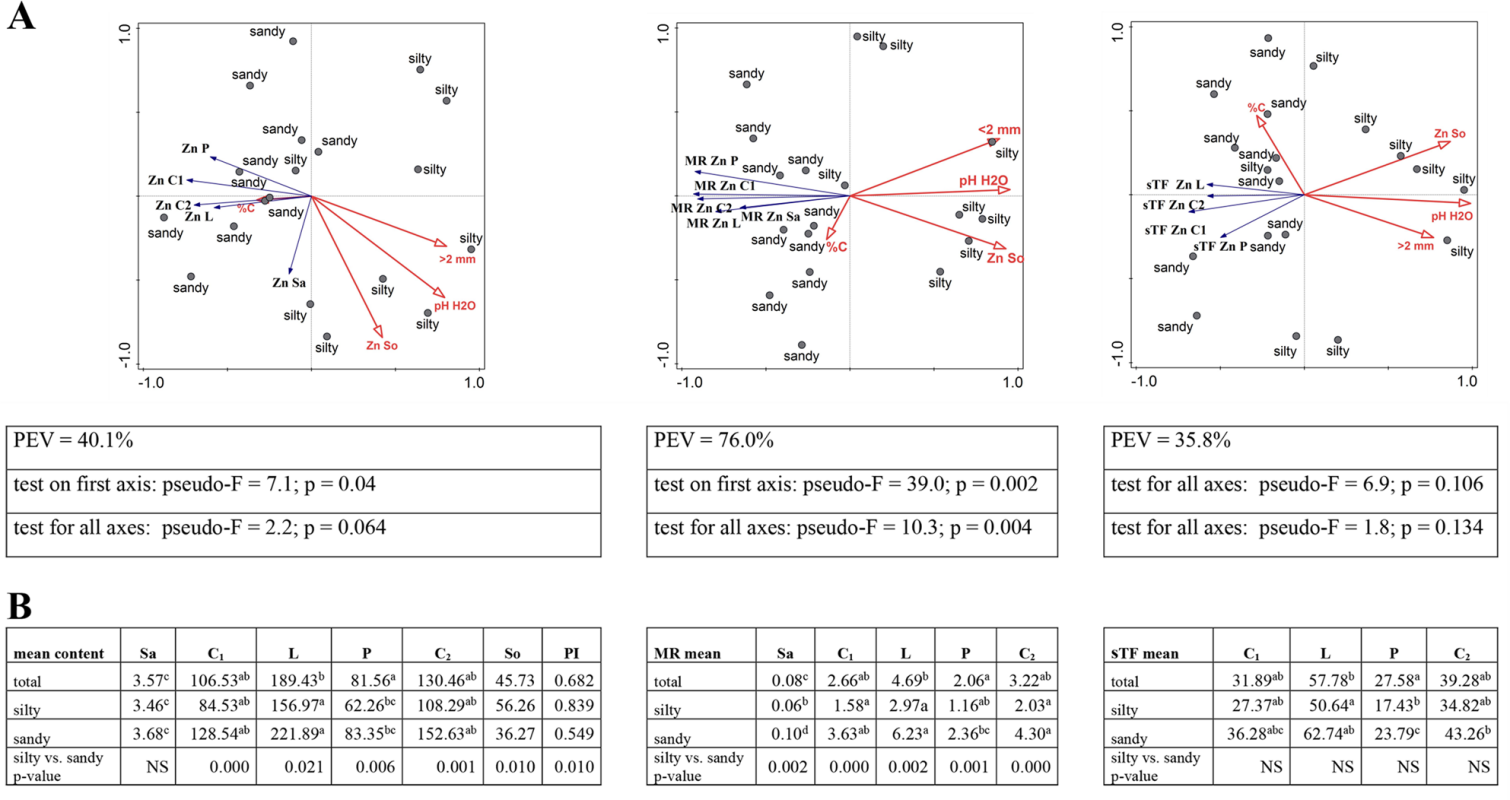
**A**-**B**. **A**—RDA triplot showing the relationships between the selected soil parameters (explanatory environmental variables) and the content of Zn in soil and plant, their mobile ratio (MR) and sap translocation factor sTF separately (the explained variables) in the context of the soil texture. In tables are shown the cumulative percentage of explained variation (PEV) and the p-value for pseudo-F which were given for four axes. **B** – the multiple comparisons of the metal content between the parts of plant organs in the context of soil texture (silty, sandy) and regardless of its type (total) and the comparison of silty vs. sandy in terms of metal content in various parts of the plant, in the soil as PI separately; the description of numbers with the same arabic letters means no statistically significant differences

**Fig. 3:**
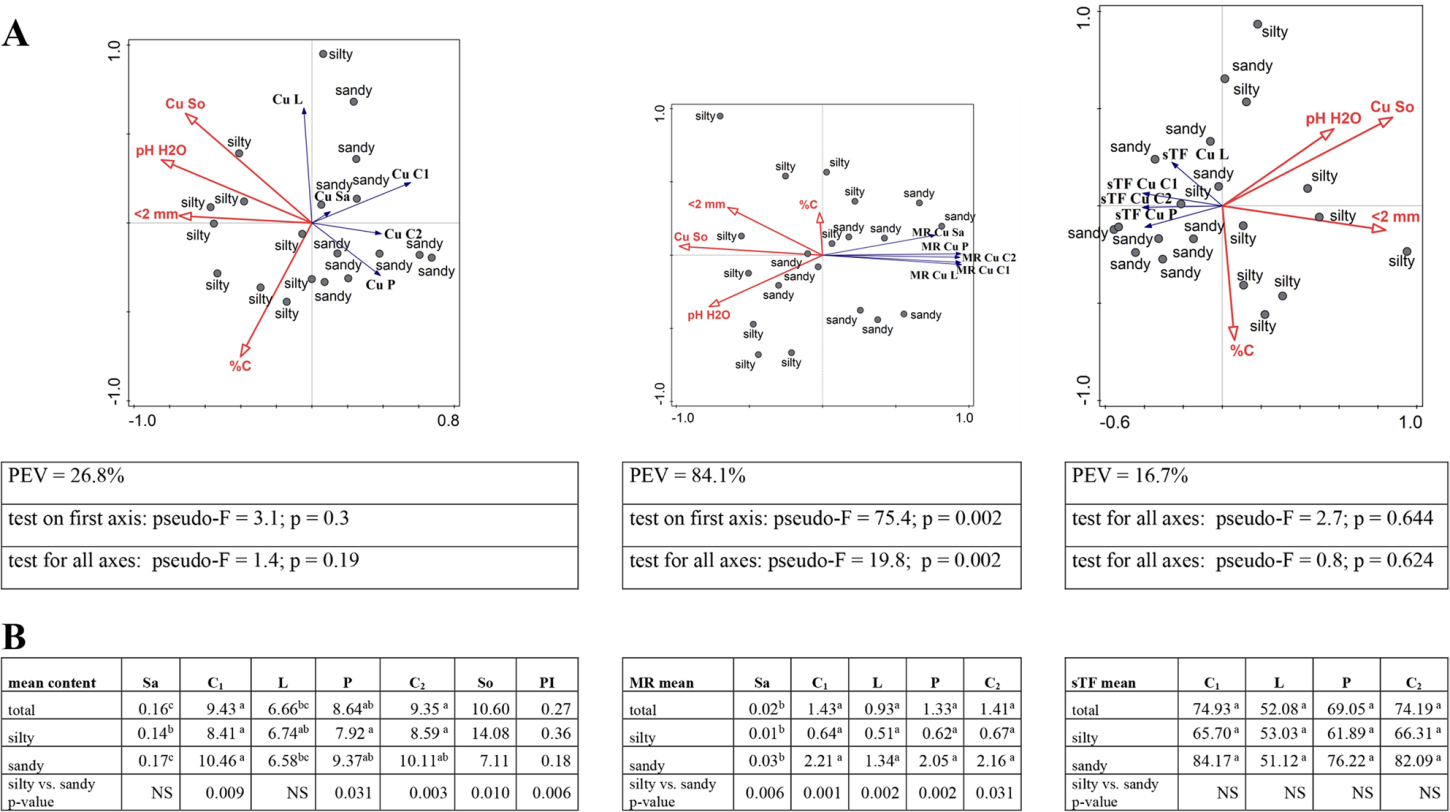
**A**-**B**. **A**—RDA triplot showing the relationships between the selected soil parameters (explanatory environmental variables) and the content of Cu in soil and plant, their mobile ratio (MR) and sap translocation factor sTF separately (the explained variables) in the context of the soil texture. In tables are shown the cumulative percentage of explained variation (PEV) and the p-value for pseudo-F which were given for four axes. **B** – the multiple comparisons of the metal content between the parts of plant organs in the context of soil texture (silty, sandy) and regardless of its type (total) and the comparison of silty vs. sandy in terms of metal content in various parts of the plant, in the soil as PI separately; the description of numbers with the same arabic letters means no statistically significant differences

**Fig. 4:**
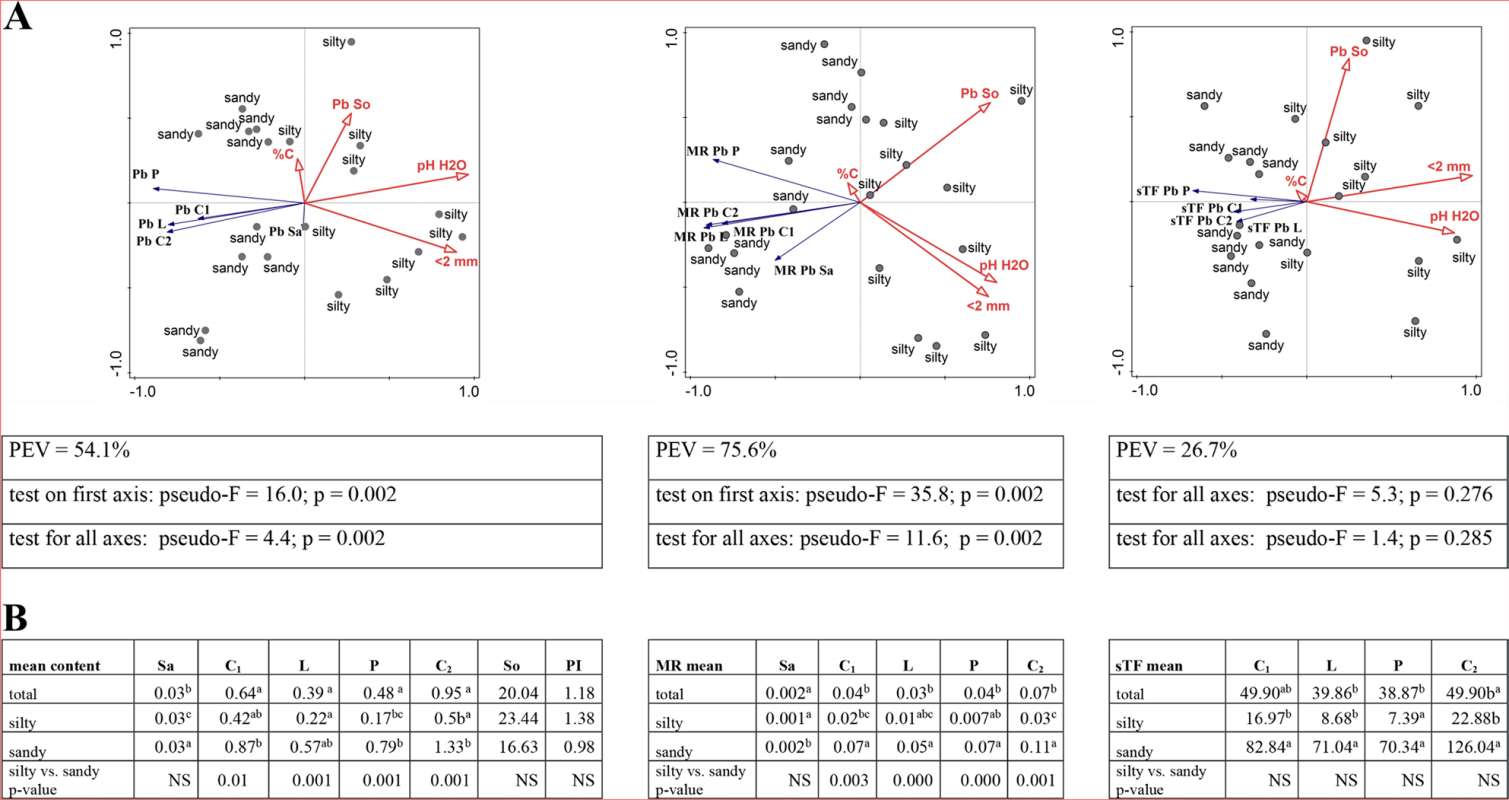
**A**-**B**. **A**—RDA triplot showing the relationships between the selected soil parameters (explanatory environmental variables) and the content of Pb in soil and plant, their mobile ratio (MR) and sap translocation factor sTF separately (the explained variables) in the context of the soil texture. In tables are shown the cumulative percentage of explained variation (PEV) and the p-value for pseudo-F which were given for four axes. **B** – the multiple comparisons of the metal content between the parts of plant organs in the context of soil texture (silty, sandy) and regardless of its type (total) and the comparison of silty vs. sandy in terms of metal content in various parts of the plant, in the soil as PI separately; the description of numbers with the same arabic letters means no statistically significant differences

**Fig. 5:**
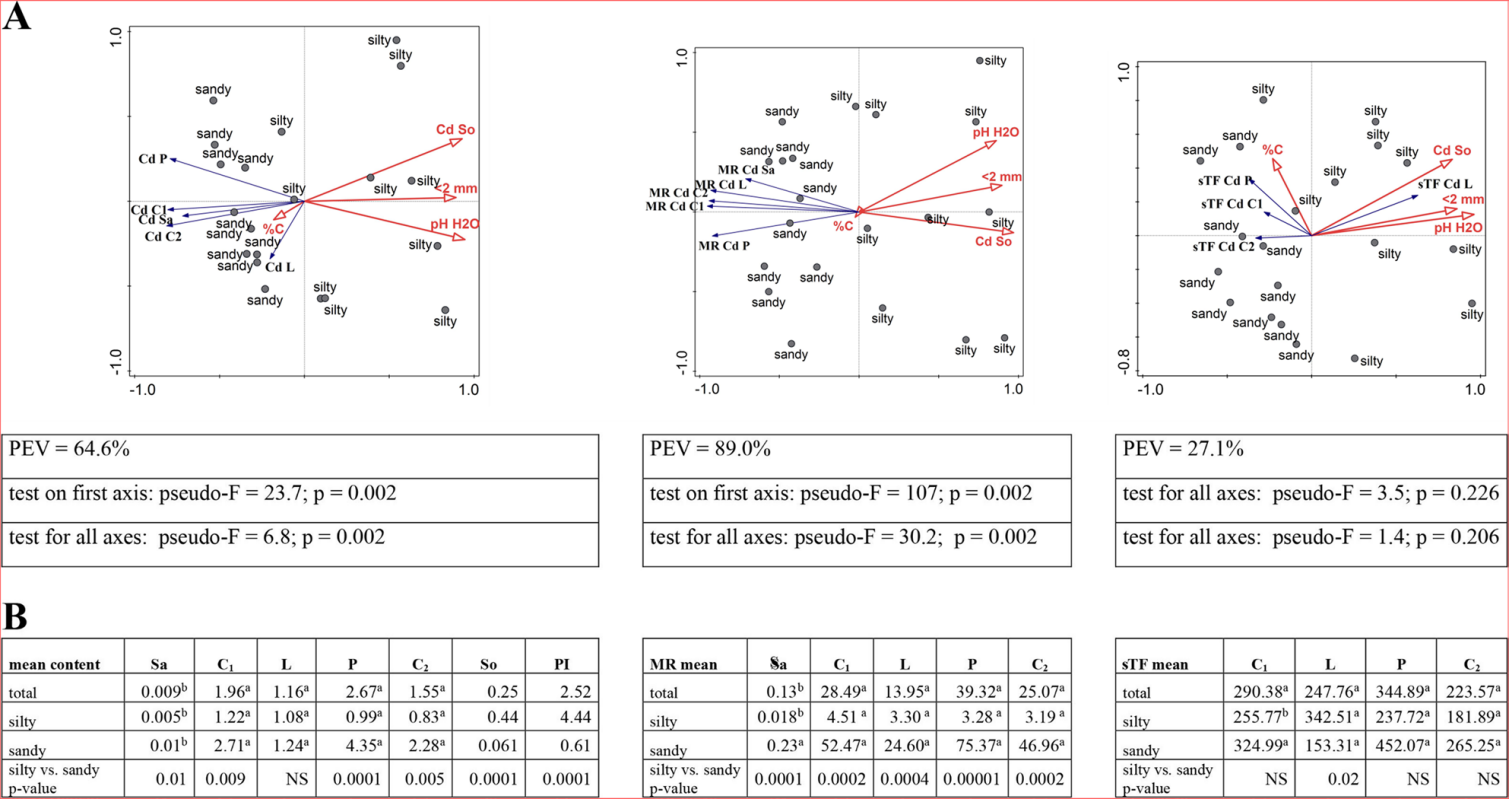
**A**-**B**. **A**—RDA triplot showing the relationships between the selected soil parameters (explanatory environmental variables) and the content of Cd in soil and plant, their mobile ratio (MR) and sap translocation factor sTF separately (the explained variables) in the context of the soil texture. In tables are shown the cumulative percentage of explained variation (PEV) and the p-value for pseudo-F which were given for four axes. **B** – the multiple comparisons of the metal content between the parts of plant organs in the context of soil texture (silty, sandy) and regardless of its type (total) and the comparison of silty vs. sandy in terms of metal content in various parts of the plant, in the soil as PI separately; the description of numbers with the same arabic letters means no statistically significant differences

**Fig. 6:**
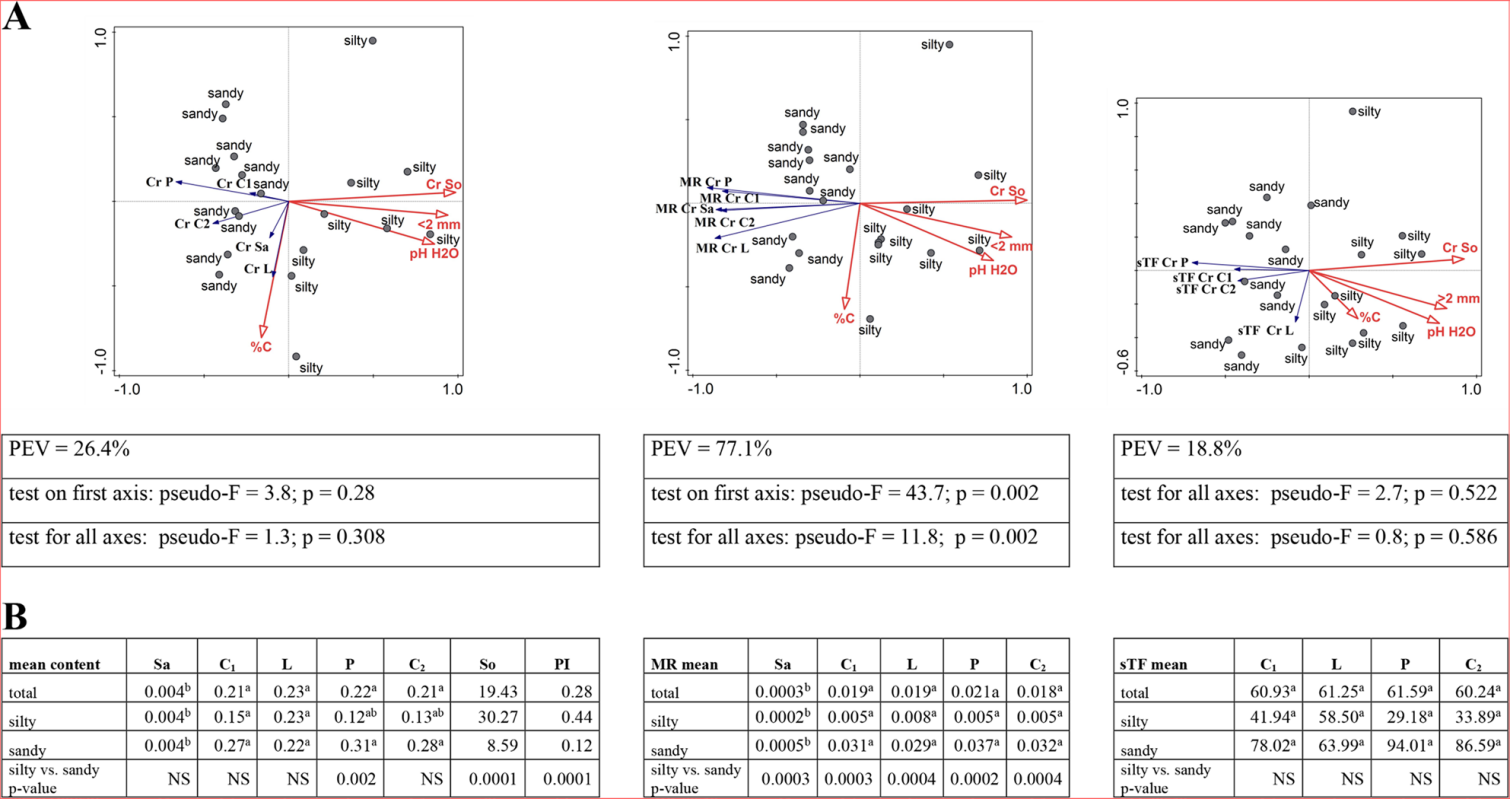
**A**-**B**. **A**—RDA triplot showing the relationships between the selected soil parameters (explanatory environmental variables) and the content of Cr in soil and plant, their mobile ratio (MR) and sap translocation factor sTF separately (the explained variables) in the context of the soil texture. In tables are shown the cumulative percentage of explained variation (PEV) and the p-value for pseudo-F which were given for four axes. **B** – the multiple comparisons of the metal content between the parts of plant organs in the context of soil texture (silty, sandy) and regardless of its type (total) and the comparison of silty vs. sandy in terms of metal content in various parts of the plant, in the soil as PI separately; the description of numbers with the same arabic letters means no statistically significant differences

**Fig. 7:**
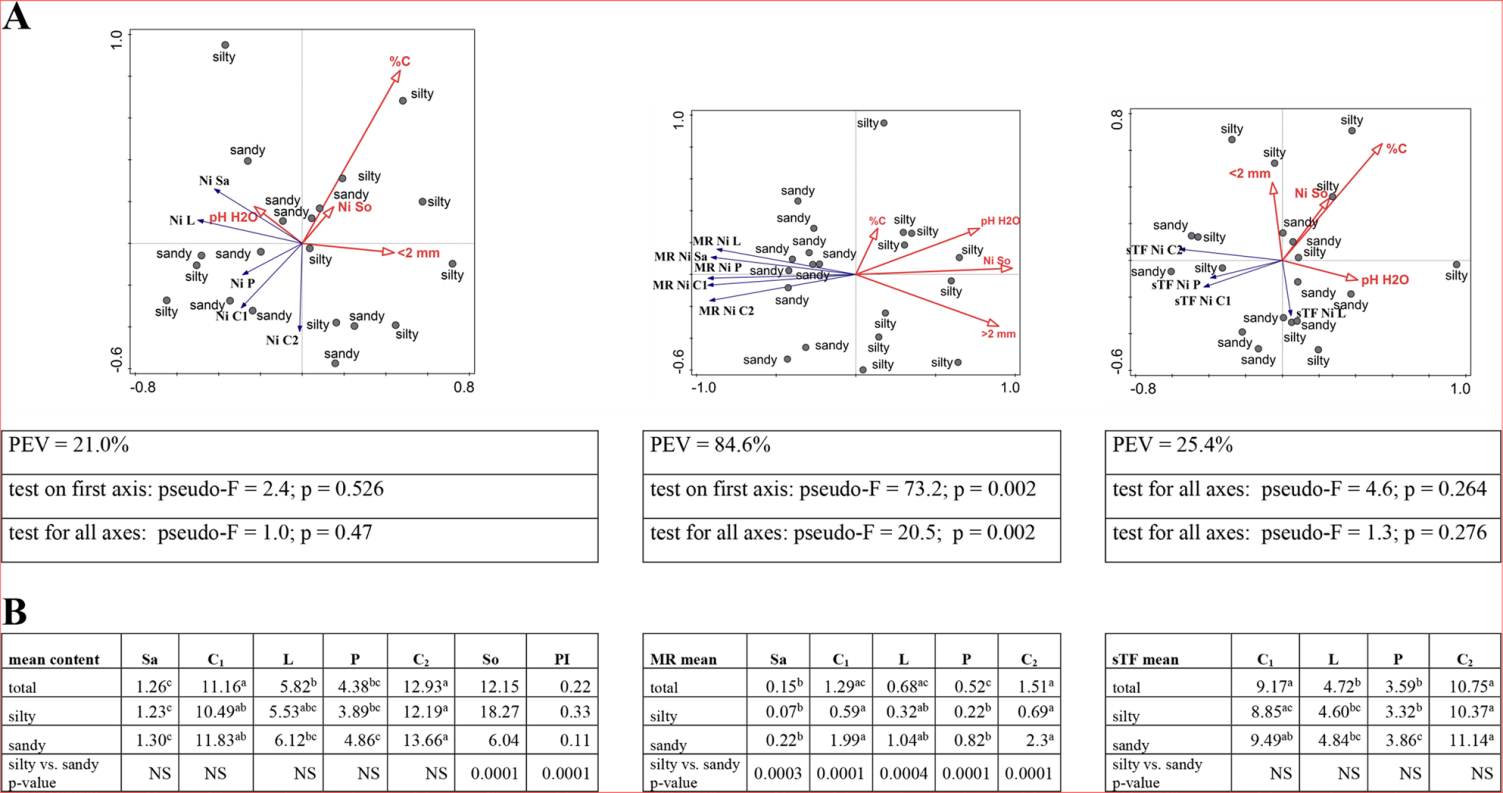
**A**-**B**. **A**—RDA triplot showing the relationships between the selected soil parameters (explanatory environmental variables) and the content of Ni in soil and plant, their mobile ratio (MR) and sap translocation factor sTF separately (the explained variables) in the context of the soil texture. In tables are shown the cumulative percentage of explained variation (PEV) and the p-value for pseudo-F which were given for four axes. **B** – the multiple comparisons of the metal content between the parts of plant organs in the context of soil texture (silty, sandy) and regardless of its type (total) and the comparison of silty vs. sandy in terms of metal content in various parts of the plant, in the soil as PI separately; the description of numbers with the same arabic letters means no statistically significant differences

The test sites differed significantly in terms of selected physicochemical properties of soils. They were divided based on soil texture, i.e. silty (RD, RZ), and sandy soils (GM, MI). The above division was reflected in a significantly lower content of soil fractions < 2 mm in sandy soils (0.8, 0.6 respectively). The second parameter differentiating the site was the pH of the soil. Based on the obtained pH measurement results in H_2_O, it was found that soils at MI and GM sites (sandy soils) differ from silty soils in their lower active acidity (4.72 and 4.95 respectively). A similar regularity can be observed by analysing the results of pH measurement in KCl, where the exchange acidity for sandy soils reached lower average values: 3.94 for MI and 4.19 for GM. Taking into account the division of sites based on soil texture, no differences were found in terms of carbon content, while the RD site was characterised by the highest C content (3.85%) compared to the others (Figs. 2A, 3, 4, 5, 6 and 7A; Additional file 1).

### The concentrations of certain heavy metals in the soil, their transport to and in the plant in the context of the physical and chemical soil parameters

#### Zinc

The zinc content in soils was one of the largest among the tested metals and ranged from 35.59 to 68.76 mg/kg. The differences between the sites were statistically significant, with lower values for MI and GM sites with sandy soils, where the zinc concentration was almost half the background value of 67 mg/kg [38, 39]. In RZ, the zinc content in the soil was very close to the value considered typical (PI Zn = 1.03) (Additional file 2). A positive relationship was shown between the zinc content of the soil and its acidity (pH H_2_O *r =* 0.664; pH KCl *r =* 0.796) and soil fraction (*r =* 0.584), while soil organic carbon was not an important parameter (Additional file 3).

In plant material, the content of this metal differed significantly statistically, and in this respect the sap was found to have the lowest concentration (Fig. 2A, B; Additional file 2). The zinc content in pollen differed significantly statistically from the content in the leaves, in which a record concentration was found, 50 times higher than in the sap. Regardless of the soil texture, the lowest contents were found in the sap, followed successively by pollen, inflorescences, and leaves (Fig. 2B; Additional file 2).

Direct ordination analysis explains the relationship between soil parameters and zinc content in plant material with more than 40% efficiency, while pointing to clear differences between silty and sandy sites. With the exception of %C, the physical and chemical properties of the soils strongly influenced the zinc content of the birch. The influence of these parameters was negative, as evidenced by long vectors with opposite returns (Fig. 2A) and correlation analysis with significant correlation coefficients for the aerial parts of birch (Additional file 3).

The transfer of zinc from the soil to the plant (MR) depended on the physicochemical characteristics of the soil. A statistically significant negative dependence on soil pH (from *r =* -0.783 for L to *r =* -0.883 for C_2_) and fraction (from *r =* -0.671 for Sa to *r =* -0.822 C_1_) (Additional file 3) was found. Birch sap was characterised by the lowest MR values (from 0.05 to 0.10); the transfer of this element to the leaves was the most intense (from 2.51 to 6.54). Regardless of the type of plant material, sandy sites (GM and MI) were characterised by the best bioavailability of this metal. RDA analysis explains very well the relationship between soil parameters and MR values for plant material. The silty and sandy sites are very well separated by the second ordinate axis (PEV = 76.0%, *p* = 0.002) (Fig. 2A; Additional file 2).

For sTF, the PEV value is very low and the probability value for the first and all ordination axes is above > 0.05 (Fig. 2A). The value of this parameter for inflorescences increases most strongly at low pH (Additional file 3). Statistical analyses have shown that the values of this indicator differ for a particular type of plant material. The lowest values were found for birch pollen and the highest for leaves without division and by soil species (Fig. 2B; Additional file 2).

#### Copper

The Cu content in the soil was the lowest at the MI site, reaching an average of 3.52 mg/kg, while at other sites the values were in the range of 10.34 – 17.82 mg/kg, with the highest for RZ (Additional file 2). The copper content in the soil was also lower than the value considered typical, i.e. 39 mg/kg [38, 39], especially at sandy sites (average PI value Cu = 0.18) (Fig. 3B). It has been shown that the content of this element in the soil significantly depends on its fraction (*r =* 0.625), and is lower on acidic soils (pH H_2_O *r =* 0.644; pH KCl *r =* 0.618). Silty sites are statistically significantly higher in Cu content than sandy sites (Fig. 3A, B; Additional file 3).

In the plant, the copper content differed significantly, with the lowest concentrations in the sap, i.e. from 0.13 in RD to 0.20 mg/kg in GM. The Cu content in inflorescences and pollen (7.90 – 10.66 mg/kg) was characterised by a tendency to accumulate the element at the GM and MI sites, with sandy soils (Fig. 3B; Additional file 2). Negative correlations with pH were found for the pre-pollination inflorescence (*r =* -0.527) and for pollen (*r =* -0.551). No similar relationships were found for leaves, and the Cu content ranged from 6.16 mg/kg in MI to 7.18 mg/kg in RZ (Additional file 2, 3). The RDA triplot indicates that the copper content in generative organs, especially in pollen and inflorescences after pollination, is lower on soils with a higher pH and a higher content of this element in the soil (Fig. 3A).

The accumulation of copper in the plant (MR) depended on the physical and chemical characteristics of the soil. A statistically significant relationship was found with pH (pH H_2_O from *r =* -0.632 for Sa to *r =* -0.731 for L) and fraction < 2 mm (from *r =* -0.476 for C_1_ to *r =* -0.686 L) (Additional file 3). The MR index was characterised by the lowest values in the sap, within the range of 0.01 – 0.05. A clearly higher accumulation was found in the leaves, i.e. MR from 0.41 to 2.00. In the generative parts of the plant, in the inflorescences and pollen, bioaccumulation was greatest, with less mobility at the RZ site and the largest in MI (Additional file 2). Regardless of the plant material, metal accumulation was statistically significantly higher at sandy sites (Fig. 3B). Direct ordination analysis explains the relationship between soil parameters and metal transport to the plant (MR index) with more than 84% efficiency indicating clear negative relationships (Fig. 3A).

Much higher values were characterised by the sTF ratio in relation to MR. Among the plant material studied, the leaves had the lowest values of this indicator (from 42.08 in GM to 60.16 in MI) and the highest in inflorescences in the range from 54.01 for P to 101.66 for C_1_ (Additional file 2). Soil textural group was not a parameter differentiating the transport of copper in the plant (Fig. 3A, B), nor was it shown that the physicochemical properties of the soil affect the volume of this transport (Additional file 3).

#### Lead

In none of the tested soil samples did the concentration of lead in the soil exceed 25 mg/kg [45], which have allowed us to conclude that the soils were not contaminated with lead. What is more, they were close to the typical ones, as indicated by the PI index values from 0.74 in GM to 1.46 in RD. The lead content in the soil was highly variable (SD standard deviation values) and ranged from 13.37 mg/kg (MI) to 24.96 mg/kg (RD). The existing differences were not statistically significant (Additional file 2). Soil texture was also not a statistically significant parameter differentiating these sites, although as indicated by multidimensional analyses at many sandy sites, the lead content in the soil is relatively low (Fig. 4A, B). A dependence of the lead content in the soil on its physicochemical parameters was also not demonstrated (Additional file 3).

With the exception of sap, the lead content of plant material and its bioavailability was strongly negatively correlated with soil pH and clay fraction (Additional file 3). The significant impact of these parameters is confirmed by RDA analyses (*p* < 0.05). For the average metal content PEV = 54.1% and for MR PEV = 75.6%. In the case of the sTF indicator, correlations with individual physicochemical parameters are much weaker or statistically insignificant, as is the result of the RDA multivariate analysis (PEV = 26.7%; *p* > 0.05) (Fig. 4A; Additional file 3).

The lowest lead content in the tested plant material was shown by sap, on average 0.03 mg/kg. In the aerial parts of the plant, lead content ranged from 0.17 mg/kg in pollen collected at silty sites to 1.33 mg/kg in inflorescences collected after pollination at sandy sites (Additional file 2). These values differed significantly statistically if the parameter grouping the site was soil texture. It can then be concluded that at sandy sites the lead content was always higher, especially in the case of generative parts (Fig. 4A, B). It is worth noting the fact that the lead content in the material collected in RZ (silty soil) is significantly lower than that collected in GM (sandy soil) (Additional file 2).

MR values indicate poor lead availability to the plant, from 0.001 for Sa in RD to 0.11 for C_1_ in MI (Additional file 2). When comparing the soil species, with the exception of sap, the availability of lead to the plant is higher at sandy sites, and is the same for generative and vegetative parts (Fig. 4A, B). At these sites, the variability of the obtained results was high (relatively high SD; Additional file 2). At the silty sites, significant differences concerned pollen and inflorescences after pollination (Fig. 4B). The accumulation of copper in the plant (MR) depended on the physical and chemical characteristics of the soil. For aerial parts of plant, a statistically significant relationship with pH (e.g. for pH H_2_O from *r =* -0.634 for C_1_ to *r =* -0.835 for P) and fraction < 2 mm (from *r =* -0.585 for C_1_ to *r =* -0.828 for P) (Additional file 3) was found. Very large fluctuations (SD) were found for the sTF index, from 2.47 for P in RZ to 185.60 for C_2_ for GM (Additional file 2). The differences between the silty/sandy sites were not statistically significant (Fig. 4B), but in many cases at the RZ position the values of this indicator were the lowest and in MI the highest (Additional file 2).

#### Cadmium

Among the elements studied, Cd was characterised by the lowest content in the soil, which ranged from 0.05 mg/kg in GM to 0.63 mg/kg in RZ, with a statistically significant trend of higher concentrations at silty sites (Additional file 2). These values depended on the chemical properties (pH H_2_O *r =* 0.738; pH KCl *r =* 0.751) and fraction of soil (*r =* 0.781) (Fig. 5A; Additional file 3). Based on the PI index, it was found that the cadmium content was on average 2.5 times higher than the background (0.1 mg/kg) [38, 39], and after taking into account soil texture, for silty soil this value was more than 4 times higher (Fig. 5B; Additional file 2).

The cadmium content of the plant depended on the chemical and physical properties of the soil. RDA analysis clarified these relationships with PEV = 64.6%. On acidic soils with a lower content of < 2 mm fraction, the content of this metal in the plant was higher, and the strongest, negative relationships occurred between pH H_2_O and the cadmium content in pollen (*r =* -0.838). The least cadmium was found in birch sap (from 0.003 mg/kg in RZ to 0.014 mg/kg in GM) and the differentiating factor was primarily soil texture. In the sap taken from the MI and GM sites (sandy sites), the Cd content was significantly higher. A similar relationship was found for generative organs, including pollen, where the cadmium content was highest (from 0.68 mg/kg in RZ to 6.53 mg/kg in MI). The leaves had a lower cadmium content (from 1.01 in MI to 1.47 mg/kg in GM) and soil species was not a differentiating factor in sites (Fig. 5A, B; Additional file 2, 3).

RDA triplot analysis for cadmium transport from soil to plant indicates that its parameters differentiate study sites very well in terms of soil species (PEV = 89.0%) and high MR values characterise sandy sites. Low pH values significantly favoured transport from the soil to the sap and aerial parts of birch, as evidenced by the values of correlation coefficients (below *r =* -0.7). The content of the < 2 mm fraction had an even stronger effect, especially for pollen (Fig. 5A; Additional file 3). The greatest mobility of cadmium from the soil was found for sites occurring on sandy soils with record bioaccumulation in pollen collected in MI (M*R =* 104.22). The accumulation of this element in the sap was the smallest, minimal in the samples collected in RZ (M*R =* 0.004) (Additional file 2).

Soil textural group was not a differentiating factor in the transport of this metal from sap to generative organs (pollen, inflorescences) and the PEV value for the RDA analysis was only 27.1% and was not statistically significant. Statistical analyses (U Mann–Whitney test) indicate better cadmium transfer to leaves at silty sites (Fig. 5A, B). In terms of this indicator, the 4 sites differed statistically significantly only for C_1_ (Additional file 2). Considering each soil parameter separately, it can be concluded that the pH and soil fraction had a statistically significant effect only on the transfer of cadmium from the sap to the leaves (Additional file 3).

#### Chromium

The average chromium content in the soil ranged from 6.85 mg/kg in MI to 38.39 mg/kg in RZ. These values were even significantly lower (more than 3 times) than the reference value (i.e. 69 mg/kg) [38, 39], and after taking into account the soil textural group, for sandy soil this value was almost 8 times lower than the background (Fig. 6B; Additional file 2). Soil species was a factor determining this parameter; at sites with sandy soils, the Cr content was significantly lower (Fig. 6B). With the exception of the organic carbon content of the soil, other physicochemical parameters significantly influenced the chromium content in it (Fig. 6A). The correlations were positive, the strongest for the soil species (*r =* 0.867) (Additional file 3).

In the plant material, the chromium content was many times lower than in the soil. Particularly low content was found in the sap (from 0.0038 mg/kg in GM to 0.0046 mg/kg in MI). Due to their large diversity (high SD), the differences between the 4 sites were not statistically significant (Additional file 2). In the aerial parts of birch, the content of Cr was similar; with the exception of pollen, differences were not noted (Fig. 6B). With the exception of pollen, the Cr content did not depend on the chemical and physical properties of the soil and soil texture, which is confirmed by statistically insignificant correlation coefficients (Additional file 3), RDA results, i.e. the arrangement and length of vectors on the triplot, PEV (26.4, *p* > 0.05) (Fig. 6A) and the U-Mann–Whitney test (Fig. 6B).

The MR indicator indicated the lowest accumulation of chromium in the sap, where it reached values from 0.0001 for RZ to 0.0007 for MI (Additional file 2). Higher accumulation was found in leaves and generative organs, and soil species influenced this parameter. At sandy sites (GM and MI), these values were statistically significantly higher than at silty sites (Fig. 6B). Chromium accumulation in the plant was higher in acidic soils and the correlation coefficients for each type of plant material were statistically significant and varied for pH H_2_O from *r =* -0.604 for C_1_ to *r =* -0.695 for P. A stronger, negative correlation was found for soil fraction with the strongest correlation for Sa (*r =* -0.827) (Additional file 3). RDA analysis confirmed these relationships with PEV = 77.1% and *p* < 0.05 (Fig. 6A).

At the studied sites, the transport of chromium in the plant (sTF) differed and concerned generative parts (Additional file 2). The average values for plant material without and with grouping by soil species did not differ statistically significantly. Confirmation of the lack of dependence is the RDA result, namely the length and position of vectors on the triplot relative to the ordinate axes, low PEV value (18.8%) and *p* > 0.05 (Fig. 6A). A negative effect of soil pH (pH H_2_O) on this indicator was found for P and C_2_ (r values of -0.658, -0.451 respectively) and clay fraction of soil only for P (*r =* -0.574) (Additional file 3).

#### Nickel

The Ni content in the soil ranged from 5.43 to 22.31 mg/kg and assumed significantly lower values at sandy sites (GM and MI). At each studied site, the nickel content in the soil was significantly lower than the geochemical background, as evidenced by low values of PI indicators; the average indicator was 0.22 (Additional file 2). As in the case of other HMs, the Ni content in the soil was positively correlated with the pH of the soil (pH H_2_O *r =* 0.758; pH KCl *r =* 0.653), it also depended positively on the content of the clay fraction (*r =* 0.817) (Additional file 3).

In the plant, the lowest nickel content was found in the sap (from 1.11 mg/kg to 1.42 mg/kg) and the highest in inflorescences (from 9.6 mg/kg to 16.03 mg/kg) (Additional file 2). As shown by statistical analyses, soil texture was not a factor differentiating sites, which is also confirmed by the RDA chart. Unlike other metals, sandy and silty sites do not cluster relative to the ordinate axes. The position of the vectors and their return indicate a negative relationship between the organic carbon content of the soil and the content of this metal in C_1_ (Fig. 7A). This is significantly confirmed by the statistical correlation coefficient (*r =* -0.486) (Additional file 3). The remaining physicochemical parameters were less important, the RDA analysis explains only 21.0% of the variability and the result is statistically insignificant (Fig. 7A).

Based on the results of the Mann–Whitney U test, it can be concluded that on sandy soils, the availability of nickel from the soil to the plant (MR) was statistically significantly higher. On the RDA triplot, these sites are clearly separated by the second axis of the ordinance. The result of this analysis is statistically significant (*p* = 0.002) with a PEV of 84.6%. The MR index reached the lowest values for the RD and RZ sites, regardless of the plant material being tested, and the highest for GM and MI. It is worth noting the highest MR values for inflorescences (average value for C_1_ – 1.29 and for C_2_ – 1.51). With the exception of %C, the physicochemical properties negatively affect the availability of nickel to the plant, which is confirmed by negative, statistically significant correlation coefficients with the strongest relationship between the clay fraction and the mobility of nickel to sap (*r =* -0.830) (Additional file 3).

The mobility of Ni in the plant (sTF) expressed by the ratio of the content of the tested metal in the aerial parts to the sap was characterised by values from 3.12 to 11.57. The GM site was characterised by the highest values regardless of the plant material studied, assuming the lowest values for P (3.59) and the highest for inflorescences (9.17 for C_1_, 10.75 for C_2_). The ordinate axes did not discriminate against the sites according to soil texture (PEV = 25.4%, *p* = 0.262) (Fig. 7A); the lack of significance of this grouping variable is confirmed by statistical analyses (Fig. 7B).

### The relationships between the content of heavy metals in the soil and the birch and their transport to and in the birch

**Fig. 8:**
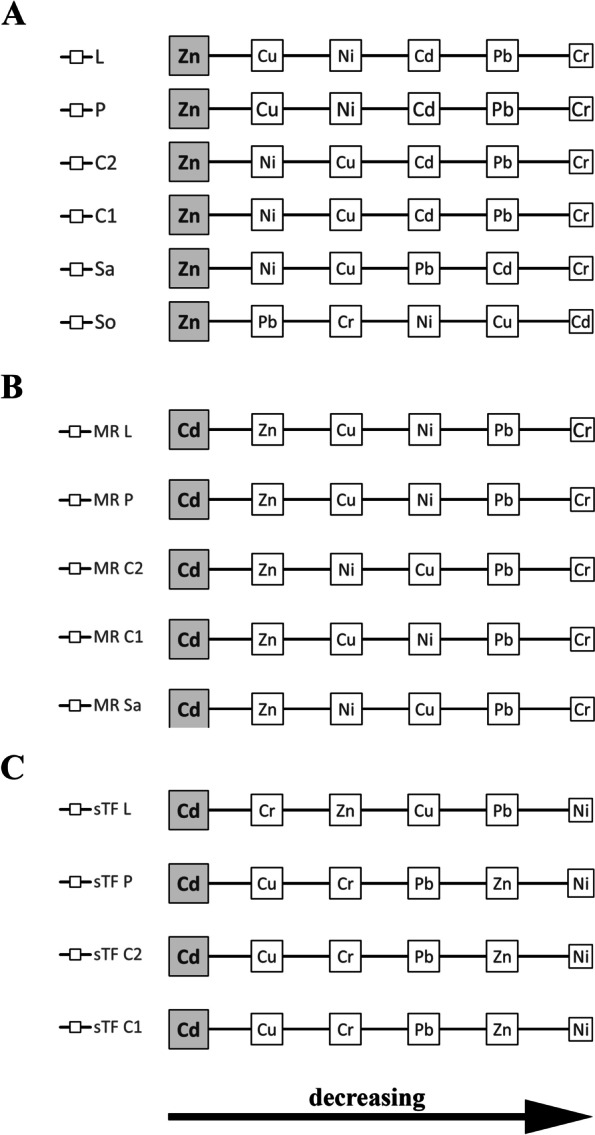
**A**-**C** The content of heavy metals in the plant and soil, their bioaccumulation (MR) and translocation (sTF) in the plant with decreasing concentration

To sum up, it should be stated that the relationships between the content of heavy metals in the soil and in different parts of birch are diverse. In the soil, the average zinc content was clearly the highest and the concentrations of the next highest elements, lead and chromium, which did not differ from each other statistically significantly, were more than twice as low. In soils, the cadmium content was very low at all sites, below 0.5 mg/kg. The sites differed in terms of the total content of heavy metals in the soil (PI total) with a clear tendency of their greater accumulation in silty soils. In plant material, zinc also reached the highest concentrations, but unlike soil, lead and chromium contents were generally the lowest (below 1 mg/kg). It should be emphasised that although the content of nickel and copper in the soil was low, in the case of plants these elements belonged to the group with the relatively highest content (Figs. 2B, 3, 4, 5, 6 and 7B; Fig. 8A; Additional file 2).

**Fig. 9:**
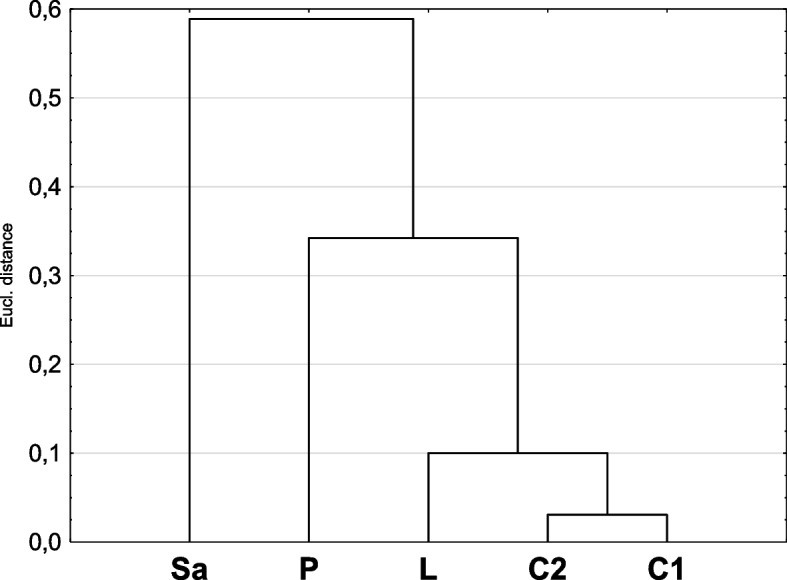
Homogeneous clusters distinguished on the basis of antioxidant properties and polyphenolic content

For each element, its content as well as the transfer from soil to plant (MR) were the smallest in the sap. The largest transfer from the soil to different parts of plants concerned cadmium (mainly catkins and pollen) and the lowest was lead and chromium, where MR did not exceed 0.1 (Figs. 2B, 3, 4, 5, 6 and 7B; Fig. 9). The transfer of elements in the plant depended on the metal, the highest was in the case of cadmium (from 233.53 for C2 to 344.8 for P) and the lowest for nickel (from 3.59 P to 10.75 C2). The sTF values for the other elements were at a similar level (from 27.58 Pb P to 74.93 Cu C1). In generative organs, the second sTF-valued element was Cu. The content of heavy elements in the soil as well as their transfer to different parts of the plant (MR) generally depended on the soil textural group in contrast to the sTF indicator indicating the transfer of elements in the plant itself (Figs. 2B, 3, 4, 5, 6 and 7B, Fig. 8B, C, Additional file 2).

#### Antioxidants

Antioxidant properties were measured by FRAP and DPPH methods and the analysis of the results indicated the highest antioxidant potential for catkins before and after pollination, while the birch sap showed the lowest antioxidant properties (Additional file 4). The analysis of the content of secondary metabolites also confirms such a diversity in the antioxidant potential of plant material. Phenolic compounds, measured using the Folin method, were the highest in catkins and also in leaves, and the lowest in birch sap. Quite low values were also observed for pollen. Leaves contained the highest content of flavonoids, while in birch sap the flavonoids content was very low (Additional file 4).

With the exception of two examples (C_2_ and the sap collected at GM), we did not observe statistically significant differences in antioxidant properties between materials sampled from different sites (Additional file 4). In several cases, the soil textural type was the factor determining the antioxidant potential measured using the DPPH, FRAP and Folin methods. According to the U Mann–Whitney, the antioxidant activity of leaves collected at sandy sites (GM and MI) was significantly higher than at silty sites (*p* = 0.014, *p* = 0.017, *p* = 0.031, respectively). Opposite relationships were found for C_2_ for the FRAP method (*p* = 0.037) (Additional file 4).

Statistically significant differences in the antioxidant properties of individual plant organs were performed by Kruskal–Wallis test (*p* < 0.000) (Table 1). The highest amounts of phenols were found in inflorescences and leaves, while sap and pollen had a significantly lower content. In terms of the content of flavonoids, three groups were distinguished. The leaves contained the highest concentrations, followed by inflorescences and pollen, and the least amount of flavonoids was found in the sap. The plant organs also differed in their antioxidant ability measured using the DPPH and FRAP methods. Regardless of the method used, two groups can be distinguished. The inflorescences and leaves were characterised by the similar highest properties, and the sap and pollen were significantly lower (Fig. 9).

**Table 1:** The homogeneous groups according to Dunn's post-hoc test

Methods	1	2	3
FRAP	C_1_, C_2_, L	P, Sa	
DPPH	C_1_, C_2_	L,P	Sa
FOLIN	C_1_, C_2_, L	P, Sa	
FLAV	C_1_, C_2_, P	C_1_, C_2_, Sa	L

The antioxidant properties are influenced by the content of metals in the soil and in the plant itself. Based on the analysis of the results of multiple regression (Table 2), it can be concluded that the metals significantly affecting the content of polyphenols and antioxidant properties are primarily copper, followed by lead, cadmium, and chromium, and occasionally nickel and zinc. An increase in the concentration of lead in the soil positively affects these properties. Zinc (negative relationship) and cadmium (positive dependence) (DPPH) content are also important variables. The cadmium content in the soil is also an important factor in enhancing the antioxidant properties of pollen (FRAP method) and an additional important explanatory variable is the content of nickel (Folin method) or copper (DPPH method). The antioxidant potential also increases with low copper content in pollen (FRAP and DPPH methods). Copper is also an important factor influencing the antioxidant properties of other parts of birch. A positive correlation was shown between the concentration of copper in the pre-pollination inflorescences and antioxidants (measured by FRAP) and the content of polyphenols (Folin method) in these organs, as well as a negative relationship with the lead content in the soil. The ability to scavenge free radicals (DPPH) in inflorescences C_1_ increases with low copper content in the soil and higher chromium content in the soil. These two variables describe these relationships to the highest degree (R^2^ = 66.5). Similarly, a negative correlation was observed between the concentration of flavonoids in C_2_ and the concentration of Cu, but the coefficient of determination was at a low level, not exceeding 20%. In leaves, a decrease in Cu concentration and an increase in lead concentration significantly affects the antioxidant properties, measured by the FRAP method, and in the case of polyphenol content (measured by Folin method), the explanatory variable is only the copper content in the leaves. These relationships are explained at 30.4% and 35.9%, respectively (Table 2).

**Table 2:** The results of regression analysis showing the relationships between the concentrations of minerals in plants and soil and antioxidant activities. The results were regression formulas with multiple correlation coefficients and multiple determination coefficients (R2). The level of significance was α ≤ 0.05

Methods /organs	Formula	R [%]	R^2^ [%]	R^2^ corr [%]
FRAP C_1_	= 21.136 **CuC1** – 122.296 **PbC1** + 115.390 ± 32.358	71.2	51.4	37.1
Folin C_1_	= 2.991 **CuC1** –16.9277 **PbC1** + 23.9971 ± 4.41	71.2	50.2	26.0
DPPH C_1_	= -83.929 **PICu** + 120.491 **PICr** + 85.447 ± 6.9453	88.1	77.6	66.5
	= -0.99088 **CuSo** + 1.89322 **CrSo** ± 6.9453	88.1	77.6	66.5
Flav C_2_	=—13.4788 **PICu** ± 1.4129	88.4	46.8	20.2
	= -1.22388 **CuSo** ± 1.7585	68.4	46.8	20.2
FRAP P	= -17.2973 **CuP** + 140.225 ± 26.567	66.0	43.6	15.4
	= 35.151 **PI Cd** + 172.511 ± 20.279	82.0	67.3	51.0
	= 3.05654 **CdSo** ± 0.71598	82.9	67.3	51.0
Folin P	= -2.60378 **CuP** + 21.1877 ± 3.9721	70.4	49.5	24.3
	= -89.0419 **PINi** + 5.1283 **PICd** + 29.4399 ± 3.5794	80.0	64.2	46.2
	= -2.52884 **NiSo** + 2.79999 **CdSo** ± 3.5794	80.1	64.2	46.2
DPPH P	= -8.51493 **CuP** + 69.7832 ± 11.537	74.2	55.1	32.6
	= 94.546 **PICu** + 15.33 **PICd** + 85.831 ± 3.1921	78.4	61.4	42.2
	= 0.96731 **CuSo** + 2.73801 **CdSo** ± 3.1921	78.4	61.5	42.2
FRAP L	= -25.551 **CuL** + 117.6013 **PbL** + 308.456 ± 32.3845	72.4	52.4	30.4
Folin L	= -3.8831 **CuL** + 51.4766 ± 4.9858	74.9	56.1	35.9
DPPH Sa	= -3.055 **ZnSa** + 507.037 **CdSa** ± 2.4908	73.1	53.5	32.0
	= 7.42735 **PIPb** ± 2.3952	73.8	54.5	31.7
	= 0.63941 **PbSo** ± 2.3952	73.8	55.5	31.7

## Discussion

The content of heavy metals in the tested soils, as indicated by the Ordinance of the Ministry of the Environment of Poland [45], did not exceed the permissible levels and did not exceed the content according to the Directive EU of 12 June 1986 [46] (Table 3). Although the individual elements were characterised by different degrees of accumulation in the soil, the content of all the heavy metals studied was within the ranges characteristic of soils of Poland according to Kabaty-Pendias and Pendias [47].

**Table 3:** Legal limit values and soil-specific ranges in Poland

Metal	Limit values (mg/kg)		Ranges for Polish soils [47]	
	Directive EU 86/278/EWG [46]	OME 2016 Poland [45]	sandy	silty
Cu	50–140	200	1–26	8–54
Zn	150–300	500	7–150	20,130
Cd	1–3	2	0,01–0,24	b.d
Pb	50–300	200	8,5–23,5	14,32
Cr	150	200	2–60	21–38
Ni	30–75	150	1–52	7–70

The division of sites based on soil species used in the work reflected the diversity of heavy metal content and lower concentration in sand composition formations, which contributed to the decision to undertake research on transport of HMs to plants.

Birch is one of the species of trees widely distributed in Poland, occurring in various habitat conditions, including on various soil species. Last research has shown that B. pendula has developed traits, which help to survive [48]. For this reason, it is used as a remedial plant [49–51], which makes it more and more widespread and conducive to use in biomonitoring [1, 51, 52]. Therefore, it becomes important to determine the influence of the soil species, as well as other physicochemical properties of the soil on the possibility of accumulation of heavy metals. As the research shows, the content of the elements studied in the work was significantly dependent on the soil species, which was particularly visible in sandy soils. According to the research, the content of clay fraction is important for the content and subsequent transport of heavy metals. This conclusion is confirmed by the literature [3, 7, 47]. It is also worth noting that the content of all the heavy metals tested was significantly lower than the given reference values used in the literature [38, 39]. According to many authors, this indicates the anthropogenic origin of the heavy metals studied [53–59]. Equally important for the transport of heavy metals to the plant are the physicochemical properties of the soil, especially pH, which at very low values affects the greater mobility of the analysed elements [5, 60]. In the conducted studies, those sites with sandy soils were characterised by lower pH values and greater bioavailability of heavy metals, which was also stated by Khan et al. [3]. As stated by Alagić et al. [5], low pH values affected the bioavailability of Cd. Migeon et al. [61], studying sites with different pH values, found changes in the bioavailability of Cd, Cr, Pb, Zn and Cu. According to Pavlović et al. [62], the diversity of soil properties can primarily affect the heavy metal content of leaves. To demonstrate the above relationships, tools can be used in the form of an MR indicator, also referred to as BAC, which is a reference of the conditions and content of heavy metals in the soil to the plant. This has been widely described in the literature and determines the content of heavy metals mainly in birch leaves to the soil [5, 62, 63]. This makes it possible to use birch as a monitoring plant and focus on the leaves as the largest accumulators, as the authors emphasise, of zinc and sometimes cadmium. Hence, most research on birch focuses on the issue of the content of heavy metals in leaves or in the roots [8, 10, 16, 17, 62, 64–72], and increasingly also sap [29, 73–76]. It is worth paying attention to the other uses of birch. A review of literature data presented by Rastogi et al. [35] indicates the widespread use of birch in medicine. The authors of the works quoted by him point to the huge wealth of biologically active substances that can be extracted from various parts of birch. Herbal materials derived from birch include bark, sap, buds, leaves, inflorescences, roots, and resin. Birch has been used among others to treat kidney stones, in stomach and liver diseases, skin diseases, urinary infection, and arthritis. Birch is also known for its antibacterial, antiviral, anticancer, immunomodulatory, and antidiabetic properties. Birch has been and is used as water/ethanol extracts, or an edible plant e.g. the pulp under the bark, phloem, leaves, buds as well as inflorescences [35, 77, 78]. From this perspective, the results determining the risk of accumulation of heavy metals in vegetative organs and in sap seem to be extremely important.

However, in the case of birch, a highly allergenic plant, attention should also be paid to the possibility of higher pollen allergenicity along with a high content of heavy metals [20]. Guedes et al. [79] found differences in protein profiles in pollen taken from contaminated sites and Depciuch et al. [80] point to structural changes in proteins contained in mugwort pollen collected in contaminated sites. From this perspective, it is important to analyse the problem of the content of heavy metals in generative organs, i.e. flowers in two developmental phases or pollen. Among the elements studied, Zn and Cd stand out, whose MR index values were higher than 1, indicating an enriched content of this metal. With the exception of sap at almost every site, these contents were higher, but only pollen collected at one site contained Cd in a dose considered toxic, i.e. 5 mg/kg. [81]. From the point of view of allergy sufferers, this is particularly dangerous because, as evidenced by Aina et al. [82], pollen taken from plants growing in contaminated areas contains more allergens that are more easily released into the environment, and has an increased ability to bind specific IgE antibodies. According to these authors soil contaminated with Cd affects the increase in expression of a protein similar to PR3 class I chitinase-like protein also in leaves. Zn, on the other hand, has a toxic dose in the range of 100–400 mg/kg d.m., and most often the accumulation of this element occurs both in leaves and generative organs, mainly from areas with sandy soils [68]. For Cu and Ni, MR values were also greater than 1 in sandy soils. Interestingly, Zn accumulated in birch leaves, which is confirmed by some authors, however, Cd, Cu and Ni have a preference for accumulation in the generative organs, catkins and pollen. The result may be among other things lower pollen viability and a decrease in germination capacity [18].

In order to determine the potential threat of heavy metals by both vegetative and generative organs of birch, an attempt was made to determine the possibility of transporting elements to individual organs. For this purpose, the TF indicator was modified, which in the literature is described as the reference of the heavy metal content in the leaves to the heavy metal content in the root of the same plant. We recognised that sap is a medium that transports elements to other organs and the content of these elements in it is the reference value. The above modification may be a more complete physiological reflection of the process of transporting and accumulating individual elements in different plant organs. TF used by other authors in the standard aboveground organ/roots arrangement, when analysing the distribution of heavy metals, indicated very diverse values, both smaller and larger than 1 [5, 51, 62, 83, 84]. The reference of the accumulation of heavy metals in individual organs to the sap caused a significant increase in the value of the new indicator, hence literature comparisons cannot be made. In different parts of the birch, the differences were indicated by the following series: Cd > Cr > Pb > Cu > Zn > Ni. The obtained sTF results for Cd confirmed high MR values, while this was not so for other tested heavy metals. The observed phenomenon indicates the risk of excessive accumulation of Cd in birch organs, with a high content in the soil, in addition, the risk of excessive accumulation of Cd increases in sandy soils with high acidification. A similar trend applies to Cr and Pb, but to a much lesser extent. It is worth noting that elements of great importance for plants, Cu and Zn, were characterised by lower values of the sTF indicator. This may actually indicate significant needs for these components, high uptake and transport, and their use, especially since they belong to the groups of elements characterised by strong (Zn) and medium uptake (Cu) [85].

Bearing in mind such a large variation in soil conditions and heavy metal content, it was decided to analyse the plant's response to changing conditions and the associated stress. One of the plants' responses to stress is the increased production of proteins involved, among others, in the chelation of metal ions and the production of pathogenesis-related proteins (PR) that defend against free radicals. It should be emphasised here that the major birch allergen, Bet v1, belongs to the pathogenesis-related protein class 10 (PR-10) family [31]. This may partly explain the increase in the incidence of inhalation allergies in urban or industrial areas, where environmental pollution is a strong stress factor [30]. Free radical production occurs continuously in all cells as part of normal cellular function. However, under biotic and abiotic stress conditions, such as heavy metal contamination, the production of reactive oxygen species (ROS) increases in the plants, resulting in induction of oxidative stress. In response to increased oxidative stress, plants start the production and accumulation of antioxidants, which significantly delay or prevent oxidative stress [86].

Antioxidants act as free radical scavengers and can thus play a significant protective role in many age-related and chronic inflammatory diseases like cardiovascular diseases, neurogenerative disorders such as Parkinson's disease, Alzheimer's disease, cancer, neurological conditions, and other diseases [87]. More than 100 disorders have been reported as ROS-mediated disorders [88]. Supplementation with exogenous antioxidants or boosting of endogenous antioxidant defences in the body have been found to be a promising method of counteracting the undesirable effects of oxidative stress. It is believed that two-thirds of the world's plant species have medicinal significance, and almost all of these have excellent antioxidant potential [86]. *B. pendula* can be counted among medicinal plants with high antioxidant potential [87]. Phenolics, which possess antioxidant properties, present in birch tissues belongs to different classes: hydroxybenozoic acids, hydroycinnamic acids and flavonoids. Among flavonoids, quercetin, myricetin, kaempferol and hyperoside predominate in birch leaves and buds [89, 90] In this study total phenolic content and one of the polyphenols class – flavonoids were analyzed.

Our results show that different organs of birch could be a good antioxidant source, especially catkins and leaves, with the highest antioxidant content. According to our best knowledge, the antioxidant potential of birch catkins before and after pollination were analysed for the first time, so we cannot compare our results to other studies. But Enayat and Banerjee [91] analysed the antioxidant potential of *Salix egyptiaca* leaves and catkins extracts and observed higher content of phenolics and antioxidants in catkins than in leaves, which is similar to our results. Comparing the content of phenolic compounds in the works of other authors, one can cite data obtained by Azman et al. [28], who detected lower phenolic content of *B. pendula* leaves, but the extraction was done with 50/50 (v/v) of ethanol. On the other hand, Penkov et al. [92] determined higher phenolic and flavonoid content (the main polyphenolic group of constituents in birch leaves) contents in leaves. However, differences in antioxidant potential may be due to the geographical origin of the trees, or the methods and solvents used for extraction. Moreover, it was proved that the antioxidant potential of leaves changes during vegetation, so the collecting period of leaf samples may be very important and may influence antioxidant potential [93, 94].

The soil species determined the antioxidant potential, and in the case of leaves collected at sandy sites it was significantly higher. This may be related to the better bioavailability of heavy metals to the plant at sandy sites. This was observed for Zn, Cu, and the highest value for Pb at sandy sites was also observed.

From the consumer point of view, the higher the antioxidant potential, the better the health-promoting properties. However, taking into account the fact that plants react to environmental stresses by increasing antioxidant content is very important in analysing heavy metal content in soil and the different organs of birch. Higher heavy metal content in the soil and organs can influence the higher antioxidant potential. On the other hand, heavy metals can be accumulated in plants and thus be introduced into our food chain. Heavy metals, such as Cd, Cu, Ni, Pb, Zn can affect plants in different ways. Some of them are necessary for the proper functioning of cellular metabolism (Cu, Zn and Ni), while others Cd, Pb, Co are not essential for the cell. Metals such as Cu and Zn are components of many enzymes or proteins. So the redistribution of metals within plants is metal specific and the elevation of levels of non-essential metals like Pb, Cd and micronutrients such as Zn and Cu may be the cause of several negative aspects of oxidative stress. Therefore, the effectiveness of a plant's antioxidant defence may be crucial for elucidating its tolerance mechanisms to heavy metals, which are common contaminants of the soil [95].

It is known that an excessively high concentration of heavy metals in the soil results in increased uptake and accumulation in the tissue of aerial organs, leading to toxic effects. According to most researchers, metal accumulation in plant tissues increases with increasing concentration in the substrate. However, the degree and rate of accumulation of individual metals varies depending on the plant species and growth stage [95]. Excess concentration of heavy metals results in ROS formation and affects the activity of enzymes involved in the basic metabolism of the plant.

We observed, in the analysis of multiple regression, that some heavy metals, especially Cu, Pb, Cd and Cr, in soil and in plant organs influence the antioxidant potential and total phenolic content of *B. pendula*. Some of them caused an increase in antioxidant potential and phenolic content. However, there is no clear response of the plant or individual organs of plants to the concentration of individual heavy metals. This may be due to the large difference in results between individual subjects. In addition, scientists observe different reactions of plants to higher concentrations of heavy metals in the soil. For example, Kulbat-Warycha et al. [95] observed that an increase in heavy metal concentrations (Ni, Cu, Zn) caused a decrease in phenol concentrations in oregano, which may have been associated with the induction of severe oxidative stress that caused oxidation of phenolic compounds included in the antioxidant system. However, Márquez-Garcia et al. [96] observed an increase in the concentration of flavonoids and polyphenols, as well as antioxidant properties in *Erica andevalensis* growing on post-mine soils contaminated with cadmium. Similarly, induction of polyphenol biosynthesis was observed in buckwheat leaves (*Fagopyrum esculentum*) sprayed with an aerosol containing nickel ions [97], which may indicate hyperaccumulation of Ni in the leaves. It must be stated that most of these experiments were conducted under laboratory-controlled conditions, where plants were cultivated in pots in soil with different concentrations of heavy metals [95–97].

## Conclusions

Along with the intensification of anthropopressure and the increase in pollution of the natural environment, there is a constant need for detailed ecotoxicological studies using modern and sensitive laboratory equipment, a constant search for various quantitative and qualitative methods of assessing the risk and consequences of toxic effects of pollutants on living organisms, and multidimensional mathematical analyses allowing for a comprehensive description of the studied phenomena. A good tool for toxicological analysis turned out to be the TF index modified by us into sTF, allowing for a numerical description of the transport of elements in the above-ground parts of plants, from sap to vegetative and generative parts.

From the point of view of human health, it is important to monitor the concentration of heavy metal ions in the soil as well as plant material that may be used and consumed by man. Among the plant materials of birch studied, sap turns out to be the safest to use for consumption. However, one should definitely have some notion of the area from which the plant material originates, taking into account the influence of soil and anthropogenic conditions, due to the eventual possibility of accumulation of Cd or Zn. When considering the aspect of silver birch usability, attention should be paid to the high content of the tested heavy metals, especially Cd in sandy soils. The obtained results indicate the need to study the impact of soil and anthropogenic conditions as factors that may promote a potential increase in the allergenic potential of birch pollen. This statement takes on an important meaning in the face of the fact that the incidence of pollen allergies is constantly increasing.

Despite significant differences in the accumulation of the studied heavy metals, none of the examined organs gave a clear response to the stress caused by their content; however, the birch reaction was evident. The obtained results lead one to wonder whether these relationships can be used in biomonitoring of environmental stress. The assessment of polyphenol content and the assessment of antioxidant potential could be a so-called screening test before further, more thorough tests aimed at searching for a specific stressor. We suggest that in the case of birch, catkins should be selected for preliminary testing, while pollen and sap would have the lowest application potential in such a procedure.

## Supplementary Information

**Additional file 1.**

**Additional file 2.**

**Additional file 3.**

**Additional file 4.**

## Data Availability

The datasets supporting the conclusions of this article are included within the article and its additional files.

## Abbreviations

HM: Heavy metal; Zn: zinc
Cu: Copper
Ni: Nickel
Cr: Chromium
Cd: Cadmium
Pb: Lead
MI: Mielec
GM: Głogów Małopolski
RZ: Rzeszów
RD: Radawiec Duży
Sa: Sap
P: Pollen
L: Leaves
C_1_: Catkins before anthesis
C_2_: Catkins after anthesis
So: Soil
MR: Mobility ratio
TF: Translocation factor
sTF: Sap translocation factor
PI_i_: Single soil pollution index
PI total: Sum of soil pollution indexes
FRAP: Ferric reducing antioxidant power
DPPH: Radical scavenging activity
RDA: Redundant analysis
PEV: The cumulative percentage of explained variation
SD: Standard deviation values
